# Metabolic reprogramming in lung cancer and its clinical implication

**DOI:** 10.3389/fphar.2024.1516650

**Published:** 2024-12-18

**Authors:** Qingqiu Huang, Lisha Fan, Mingjing Gong, Juntong Ren, Chen Chen, Shenglong Xie

**Affiliations:** ^1^ Department of Thoracic Surgery, Sichuan Provincial People’s Hospital, University of Electronic Science and Technology of China, Chengdu, China; ^2^ Department of Pulmonary and Critical Care Medicine, Sichuan Provincial People’s Hospital, University of Electronic Science and Technology of China, Chengdu, China; ^3^ Sichuan Province Revolutionary Disable Armyman Rehab, The First Veterans Hospital Of Sichuan Province, Chengdu, China; ^4^ School of Pharmaceutical Sciences, Guizhou University, Guiyang, China; ^5^ Department of Outpatient, Sichuan Provincial People’s Hospital, University of Electronic Science and Technology of China, Chengdu, China

**Keywords:** metabolic reprogramming, lung cancer, glucose, lipid metabolism, amino acid metabolism

## Abstract

Lung cancer has posed a significant challenge to global health, and related study has been a hot topic in oncology. This article focuses on metabolic reprogramming of lung cancer cells, a process to adapt to energy demands and biosynthetic needs, supporting the proliferation and development of tumor cells. In this study, the latest studies on lung cancer tumor metabolism were reviewed, including the impact of metabolic products and metabolic enzymes on the occurrence and development of lung cancer, as well as the progress in the field of lung cancer treatment targeting relevant metabolic pathways. This provides some promising potential directions into exploring lung cancer tumor metabolism and helps researchers to better understand lung cancer.

## 1 Introduction

In 2012, 14.1 million new cases of malignant tumors were diagnosed globally and 8.2 million people died from malignant tumors. The most commonly diagnosed types of cancer inlude lung (1.82 million cases), cancer (1.67 million cases), and colorectal (1.36 million cases); cancer (1.6 million deaths), cancer (745,000 deaths), and cancer (723,000 deaths from) were the leading causes of cancer-related deaths (Ferlay et al., 2015). Lung cancer poses a significant challenge to global health. Approximately 2.1 million people are diagnosed with lung cancer each year, and about 1.8 million people lose their lives as a result (Fr et al., 2017; Bray et al., 2018; Muscat et al., 1997). The global incidence of cancer continues to rise, despite a decline in the incidence of cancer in men in some Western countries. The diagnosis of lung cancer relies on histological examination, immunohistochemistry, and molecular analysis. Among all lung cancer cases, non-small cell lung cancer (NSCLC) accounts for about 85%, while small cell lung cancer (SCLC) accounts for about 15%. The 5-year survival rate for cancer patients usually ranges from 15%–20%. The 5-year survival rate for patients with non-small cell lung cancer can reach 90% in stage 1A, but drops to less than 10% by stage 4. For patients with small cell lung cancer, the 5-year survival rate for limited-stage small cell lung cancer is about 30%, while the 5-year survival rate for extensive-stage small cell lung cancer is less than 10%. Treating lung cancer requires a multidisciplinary team effort, including surgery, radiation therapy, systemic therapies (e.g., chemotherapy, targeted therapies, immune checkpoint inhibitors), and supportive care including palliative care (Zappa and Mousa, 2016). Developing a treatment plan requires consideration of tumor characteristics, staging, and patient-specific circumstances. Although the rapid development of targeted therapies and immune checkpoint inhibitors has brought new hope for the treatment of lung cancer, lung cancer remains a clinical challenge that is difficult to cure completely. Therefore, the development of new treatment strategies is a key focus of current lung cancer research.

Cellular metabolism is essential for maintaining homeostatic redox balance and providing the necessary energy and substrates to support physiological functions in the human body. In the context of oncogenesis and tumor progression, cellular metabolism undergoes significant alterations to fulfill the bioenergetic and biosynthetic requirements of the unchecked proliferation of malignant cells (Ward and Thompson, 2012; Mg et al., 2009; Hanahan and Weinberg, 2011). A notable example of this phenomenon is the upregulation of glycolysis under aerobic conditions, known as the Warburg effect, which is observed in many cancer cells. Although the manifestation of tissue heterogeneity varies among different cancer types, a subset of common metabolic modifications is consistently observed, characterizing a fundamental aspect of cancer cell biology. This metabolic reprogramming is now recognized as one of the hallmarks of cancer (Hanahan, 2022; Pavlova et al., 2022; Kroemer and Pouyssegur, 2008). Here, our comprehension of the intricate mechanisms underlying tumor metabolic reprogramming remains incomplete. Therefore, there is a pivotal need to delineate the molecular underpinnings of these metabolic shifts since it is indispensable for developing novel therapeutic strategies targeting cancer cell metabolism.

In this work, advanced researches related to tumor metabolism in lung cancer were summarized, in which we briefly outlined the impact of metabolic products and enzymes on the occurrence and development of lung cancer, the predictive value of related metabolic products and enzymes for lung cancer prognosis, and the progress of targeting related metabolic pathways in the field of lung cancer treatment. This study provides some promising potential directions related to tumor metabolism in lung cancer research, helping researchers to better understand lung cancer.

## 2 Metabolic reprogramming in tumor

**FIGURE 1:**
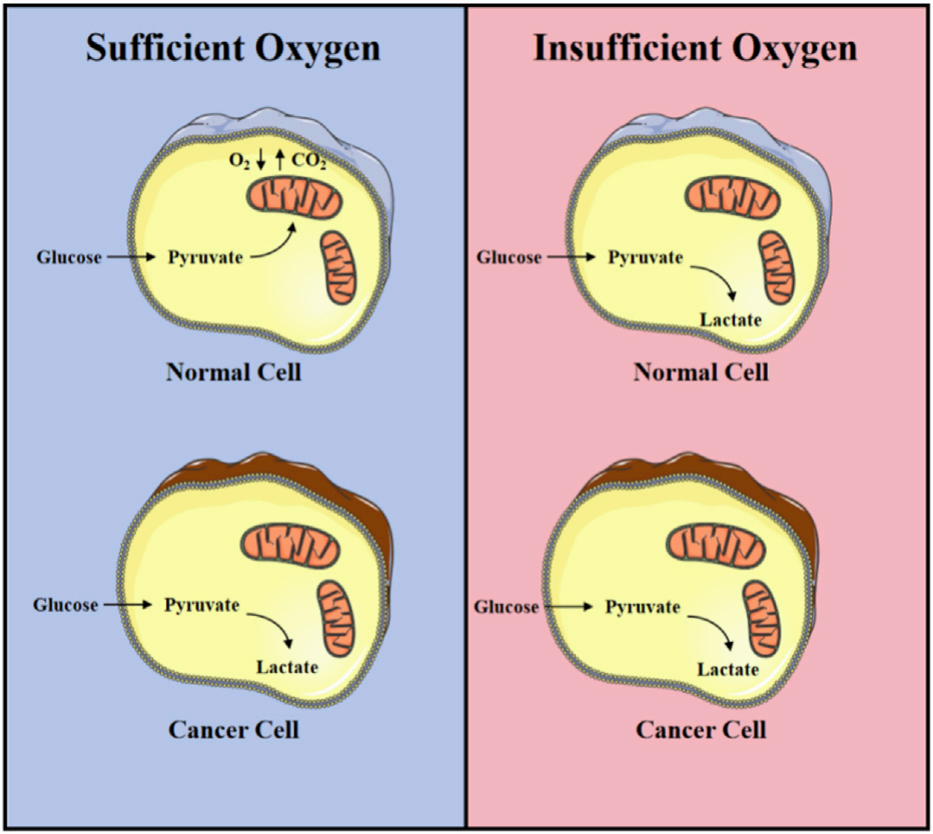
Warburg effect: Differences in energy metabolism between normal nuclear lung cancer cells under aerobic and hypoxic conditions.

Tumor cells differ from normal tissue cells in their metabolic processes for energy and biomolecules during growth and proliferation, and this field has become a new hot topic in oncology research. Research on tumor metabolism can be traced back to the 1920s when German biochemist Otto Warburg first described that tumor cells tend to produce energy through glycolysis, even in the presence of sufficient oxygen, a phenomenon later known as the “Warburg effect” (Koppenol et al., 2011; Icard et al., 2018; Vaupel and Multhoff, 2021) (Figure 1). Tumor metabolic reprogramming refers to the process by which cancer cells regulate their own metabolic processes in various ways to meet the energy demands of rapid proliferation. This mode of metabolism is different from that of normal cells, which rely more on the process of oxidative phosphorylation in mitochondria for energy production. Over time, researchers have found that tumor metabolism involves the changes in energy production and the metabolic reprogramming of glucose, amino acids, fatty acids, and nucleotides, which support the rapid proliferation and survival of tumor cells. Tumor cells alter metabolic pathways to meet their needs for biosynthetic precursors, such as increasing the expression of certain metabolic enzymes, changing the regulation of metabolic pathways, and interacting with other cells in the tumor microenvironment.

Research on tumor metabolism not only helps us to better understand tumor biology but also provides new strategies for tumor diagnosis, prognosis, and treatment. For example, by targeting tumor-specific metabolic pathways, new anticancer drugs can be developed that selectively inhibit the metabolic activity of tumor cells with minimal impact on normal cells. In addition, changes in tumor metabolic products can serve as the biomarkers for early detection of tumors and monitoring treatment effects.

## 3 Glucose metabolism in lung cancer

Glucose metabolism is fundamental to cellular function and is crucial for maintaining cell proliferation and determining cell fate (Zhu and Thompson, 2019). It not only provides the energy required by cells but also supplies the necessary carbon skeletons for cellular biosynthetic processes. Glucose, as the primary source of energy, is converted into pyruvate through the glycolytic process within the cell, and then under aerobic conditions, it enters the mitochondria for oxidative phosphorylation, producing approximately 30–32 molecules of ATP per glucose. In addition, glucose also provides the carbon precursors needed for synthesizing nucleic acids, fatty acids, and phospholipids in cell membranes through the pentose phosphate pathway and the serine synthesis pathway (Arren et al., 2012; Mulukutla et al., 2016; Mergenthaler et al., 2013). Even when oxygen is abundant, tumor cells tend to produce energy through the glycolytic process, which can rapidly produce a large amount of intermediates required for biosynthesis, with an appropriate ATP/ADP ratio. Hence the proliferation and malignant progression of tumor cells are more dependent on sugar metabolism (Walker-Samuel et al., 2013; Boroughs and DeBerardinis, 2015; Qin et al., 2020).

**FIGURE 2:**
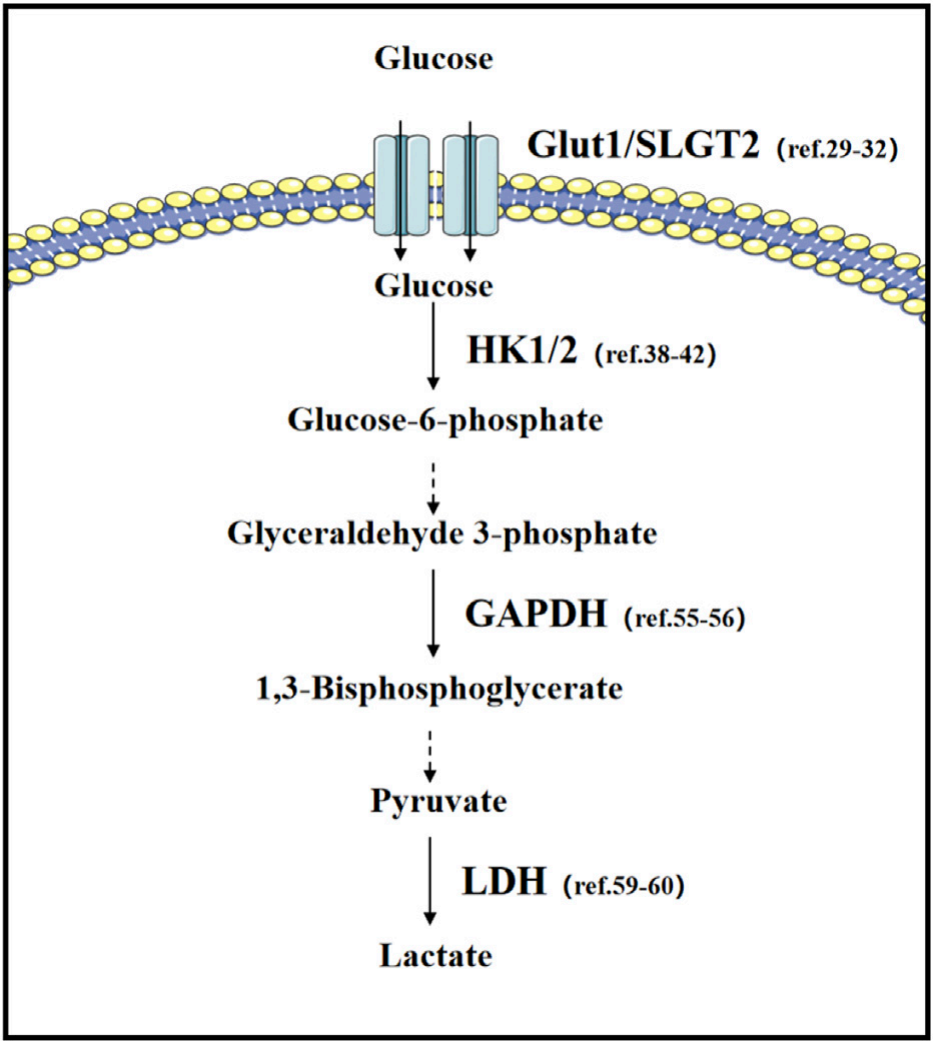
Glucose metabolism in lung cancer: important transporters and rate-limiting enzymes in glucose metabolism, as well as relevant literature in lung cancer research.

The metabolic diversity of tumor cells is perfectly demonstrated in lung cancer (Han et al., 2023; Chen et al., 2019) (Figure 2). In non-small cell lung cancer, Transforming Growth Factor (TGF-β) has a dual function in regulating glycolysis: under normal oxygen levels, TGF-β can inhibit glycolysis, but under hypoxic conditions, both *in vitro* and *in vivo*, TGF-β significantly promotes tumor cell glycolysis. This transition is due to the binding of Hypoxia-Inducible Factor (HIF-1α) to the MH2 domain of phosphorylated Smad3, leading to changes in Smad partners under hypoxic conditions (Huang et al., 2021).

### 3.1 Glucose transporters

Glucose transporters (GLUTs) are a group of proteins located on the cell membrane that are responsible for transporting glucose from the bloodstream into the cell. Glucose is the primary energy source for cells and is essential for sustaining life activities. Dysfunction of glucose transporters is associated with a variety of diseases, including diabetes, cardiovascular diseases, and cancer. The role of GLUTs in cancer is extremely important as they are responsible for transporting glucose from the blood into cells, providing the necessary energy for the rapid proliferation of cancer cells. In many types of cancer, such as lung, liver, stomach, breast, ovarian, and colorectal cancers, the expression levels of GLUT1 are increased (Yadav et al., 2024; Zambrano et al., 2019; Macheda et al., 2005). This increased expression allows cancer cells to take up a large amount of glucose to maintain their growth and development; therefore, GLUT1 can serve as a marker for detecting cancerous changes and is used in clinical examinations (Barron et al., 2016). Studies have shown that in non-small cell lung cancer, anti-Glut1 antibodies can inhibit proliferation and also enhance the anti-proliferative effects of cisplatin, paclitaxel, and gefitinib (Rastogi et al., 2007). Certain types of lung cancer become refractory due to specific genetic changes, such as KEAP1-mutated lung cancer, which is resistant to most current treatment methods. However, research has found that the lack of KEAP1 promotes the dependency of lung cancer cells on glucose, and KEAP1-mutated/deficient lung cancer cells are more sensitive to glucose deprivation than their wild-type counterparts. Therefore, targeting metabolism may be a better solution for specific types of refractory lung cancer (Koppula et al., 2021a; Saggese et al., 2024). Sodium-Glucose Transport Protein 2 (SGLT2) is another type of glucose transport protein that primarily functions in the kidneys. For example, SGLT2 inhibitors can improve the overall survival rate of non-small cell lung cancer patients with pre-existing diabetes (Luo et al., 2023).

### 3.2 Glycolysis-related enzymes

Hexokinase (HK) is the enzyme that catalyzes the conversion of glucose to glucose-6-phosphate, the initial step of glycolysis and the rate-limiting step of the pathway. There are four hexokinase isoforms in the human body: HKI, HKII, HKIII, and HKIV. Among these isoforms, HKII has garnered significant attention due to its abnormally elevated expression levels in a variety of cancers, which is closely associated with the metabolic reprogramming and progression of tumors (Roberts and Miyamoto, 2015; Li R. et al., 2022; DeWaal et al., 2018; Ciscato et al., 2021; Lee et al., 2019). Specifically, the overexpression of HKII has been linked to the glycolysis-dependent metastasis of lung cancer cells (Wiel et al., 2019). Also, it is reported that HKII regulates proliferation of lung cancer cells and predicts poor prognosis in lung cancer patients (Duan et al., 2019; Yu et al., 2024; Yang L. et al., 2021). Those finding provides a theoretical basis for therapeutic strategies targeting HKII. Moreover, studies have identified selective inhibitors of HKII, which have shown significant effects *in vivo*, substantially suppressing the glycolytic process and cancer cell proliferation in lung cancer cells, with high inhibitory potency and low toxicity. Therefore, these HKII inhibitors are considered promising candidate drugs for future lung cancer therapy and are worth further development and research (Zhang et al., 2021).

Lactate Dehydrogenase (LDH) is a class of enzymes widely present in various human tissues, playing a crucial role in the glycolysis process, particularly under anaerobic conditions, by catalyzing the conversion of pyruvate to lactate (Sharma et al., 2022). The role of LDH is especially significant in the occurrence and development of cancer. Cancer cells tend to produce energy through the glycolytic process, and LDH converts pyruvate to lactate in this process, promoting the proliferation and survival of cancer cells (Claps et al., 2022; Certo et al., 2021; Doherty and Cleveland, 2013). The expression patterns and roles of the two subunits, LDHA and LDHB, in tumors are not entirely the same. LDHA is overexpressed in many malignant tumors and is associated with tumor growth, maintenance, and invasion. The role of LDH in the tumor microenvironment is not limited to energy metabolism; the lactate it produces can promote, invasion (Dong et al., 2017; Brand et al., 2016), and metastasis (Dong et al., 2017), and may also affect immune responses (Brand et al., 2016; Xu et al., 2021). In addition, the expression level of LDH is closely related to the prognosis of cancer patients, with high levels of LDH usually being associated with poor prognosis (Zhang et al., 2016; Gu and Yang, 2023; Chen L. et al., 2024). Studies have found that 90% of patients diagnosed with non-small-cell lung cancer express LDH, which is not present in non-cancerous tissues. Analysis shows that LDH is also crucial for predicting lung cancer prognosis (Mezquita et al., 2018; Mathur et al., 2023); high levels of LDH in the serum of NSCLC and small-cell lung cancer patients before treatment are associated with lower survival rates, and LDH levels in NSCLC patients’ serum are negatively correlated with progression-free survival. Thus, high LDH expression in lung cancer is linked to poor prognosis and may be related to poor responses to standard chemotherapy (Zhang et al., 2016).

Glyceraldehyde-3-phosphate dehydrogenase (GAPDH) catalyzes the conversion of glyceraldehyde-3-phosphate into 1,3-bisphosphoglycerate, a key step in the metabolic pathway that generates energy for cells (Colell et al., 2009). GAPDH is commonly overexpressed in various cancers, indicates a poor prognosis, and is linked to immune cell infiltration and the expression of immune checkpoint genes (Wang J. et al., 2023). Studies have shown that GAPDH plays a crucial regulatory role in linking energy metabolism with the cell cycle network. Inducing senescence in LKB1-deficient non-small cell lung cancer cells through GAPDH depletion suggests a novel strategy for controlling the proliferation of tumor cells (Phadke et al., 2011). Furthermore, in the study of lung adenocarcinoma, bioinformatics analysis has revealed GAPDH as a prognostic marker associated with ferroptosis, whose expression level correlates with the tumor’s immune microenvironment. Combining immunotherapy with strategies targeting GAPDH to induce ferroptosis in lung adenocarcinoma may offer a novel therapeutic approach (Ouyang et al., 2023).

## 4 Lipid metabolism in lung cancer

Fatty acid metabolism plays a crucial role in the occurrence and development of cancer. Cancer cells tend to meet their increased demand for energy and biosynthetic precursors by enhancing fatty acid synthesis and oxidation. Key enzymes in fatty acid synthesis, such as Fatty Acid Synthase (FASN), Acetyl-CoA Carboxylase (ACC), and ATP Citrate Lyase (ACLY), are upregulated in various cancers and are closely associated with tumor progression (Paul et al., 2022; Menendez and Lupu, 2017; Menendez and Lupu, 2007; Wang Y. et al., 2022; Yu et al., 2023; Wang et al., 2015; Khwairakpam et al., 2015; Merino et al., 2017). Fatty acid metabolism not only affects the energy production of cancer cells but may also impact the tumor microenvironment and immune response. For instance, NADPH generated during fatty acid oxidation helps maintain redox balance, while intermediates produced in fatty acid synthesis can act as signaling molecules affecting cell proliferation and survival (Ju et al., 2020). In cancer therapy, targeting fatty acid metabolism offers a new strategy for cancer treatment. For example, inhibiting FASN can reduce the proliferation and survival of cancer cells, and FASN inhibitors have shown anti-tumor effects in preclinical models (Lupu and Menendez, 2006; Wang H. et al., 2022; Singha et al., 2020). Additionally, key enzymes in fatty acid oxidation (FAO), such as Carnitine Palmitoyltransferase 1 (CPT1) and Cyclooxygenase-2 (COX-2), also play significant roles in tumor growth and progression (Hu et al., 2023; Liu Z. et al., 2023; Wang et al., 2018; Liao et al., 2007; Evans and Kargman, 2004; Ma et al., 2024). Overall, fatty acid metabolism is vital for the occurrence, development, and metastasis of tumors and may become a new target for cancer therapy. Future research needs to be conducted to further explore the specific mechanisms of fatty acid metabolism in different types of cancers and develop targeted treatment methods.

### 4.1 Fatty acid transporter

CD36 is a scavenger receptor expressed in various cell types, involved in multiple processes such as lipid uptake, immune recognition, inflammatory responses, molecular adhesion, and cellular apoptosis. It plays an important role in the treatment of blood disorders and cancer, making it a potential therapeutic target (Abumrad et al., 2021). There is a high expression level of CD36 in tumor cells, and its expression in metastatic foci is often more significant than that in the primary tumor site. This finding suggests that CD36 plays an important role in the development of tumors and may indicate a poor prognosis (Yang et al., 2024; Ww et al., 2023; Wang and Li, 2019). Overweight and lipid metabolism disorders have become increased risk factors for lung cancer (Nath and Chan, 2016; Hale et al., 2014; Pascual et al., 2017). Studies have confirmed that palmitic acid (PA) or high-fat diet (HFD) promote the proliferation and metastasis of lung adenocarcinoma cells in a CD36-dependent manner (Li et al., 2023; Liu L-Z. et al., 2023). Additionally, CD36 is also associated with the functional impairment of immune cells in the lung cancer tumor microenvironment. CD36-positive CD8-positive T cells exhibit compromised immune functions, and a high level of infiltration of CD36-positive CD8-positive T cells predicts poor prognosis and inferior response to chemotherapy in patients with non-small cell lung cancer (Ao et al., 2023). These research efforts have provided new insights into lung cancer research, suggesting that CD36 is a valid target for Lung Adenocarcinoma therapy (Bai et al., 2013; H et al., 2024).

**FIGURE 3:**
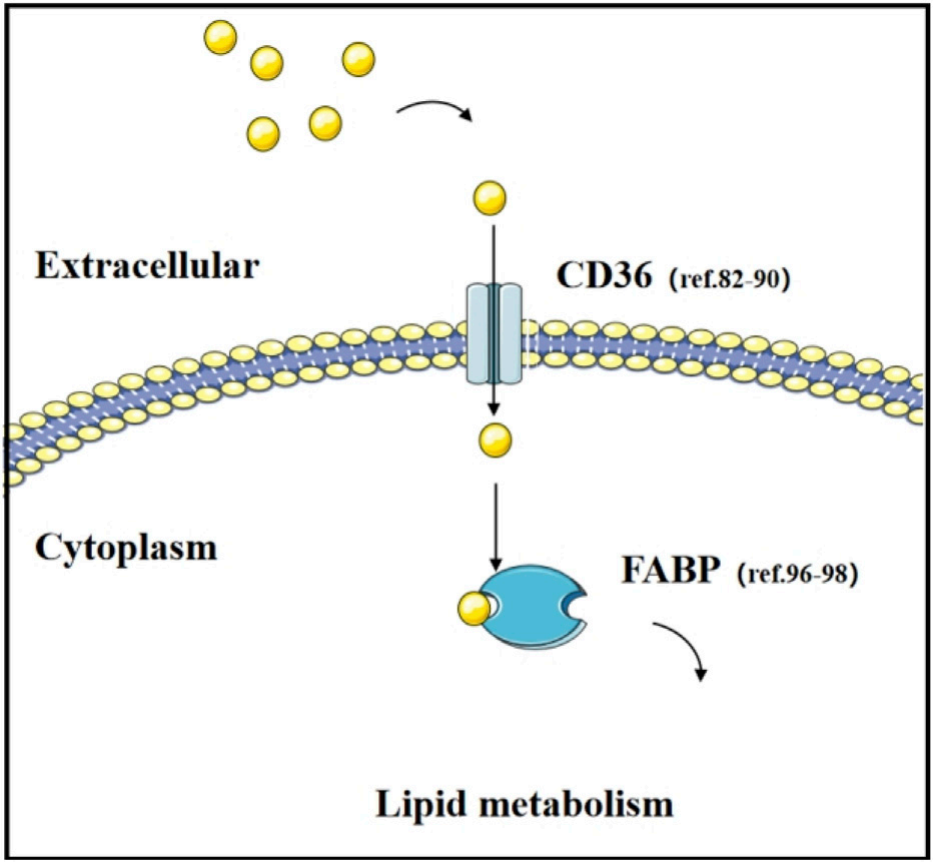
Transport of fatty acid and their transporters. The function of fatty acid transporters (CD36 and FABP) and relevant literature in lung cancer research.

Fatty Acid Binding Proteins (FABPs) are members of the lipid-binding protein superfamily with low molecular weights (14–15 kDa). They are expressed in various tissues and play crucial roles in the metabolism, transport, and signaling of fatty acids (Li et al., 2020). In cancer, the aberrant expression of FABPs has been found to be closely associated with the occurrence and progression of tumors (Greenhill, 2018; Elsherbiny et al., 2013; Nimptsch et al., 2023). For instance, the expression of FABP1 is reduced in hepatocellular adenomas (HCA). This phenomenon is linked to the decreased expression of hepatocyte nuclear factor-1α (HNF-1α), a transcription factor that plays a central role in regulating hepatocyte growth and differentiation. In the absence of FABP1, an increase in the levels of polyunsaturated fatty acids and a decrease in saturated fatty acids are observed. Concurrently, changes in the levels of prostaglandins such as prostaglandin E2, which is regulated by FABP1, are associated with the occurrence and progression of colorectal cancer. These findings reveal the potential role of FABP1 in the development of liver tumors and highlight the significance of FABPs in cancer metabolism (McKillop et al., 2019). In a mouse model of lung cancer metastasis, FABP5-deficient mice were found to be more susceptible to tumor metastasis (Yang S. et al., 2021; Liu et al., 2013). Mutations in the Src gene are common in lung cancer, and the release of Src’s inhibitory effect leads to an increase in FABP4 levels. This elevated expression of FABP4 is closely associated with the decrease in lipid droplets and a slowdown in tumor growth. Furthermore, in paired samples from lung cancer patients, the upregulation of FABP4 has also been confirmed to correlate with better patient prognosis (Hua et al., 2019). These studies not only reveal how tumor cells can promote cancer progression by regulating metabolism but also provide new insights into the role of metabolic reprogramming in tumor development (Figure 3).

### 4.2 Fatty acid synthesis

**FIGURE 4:**
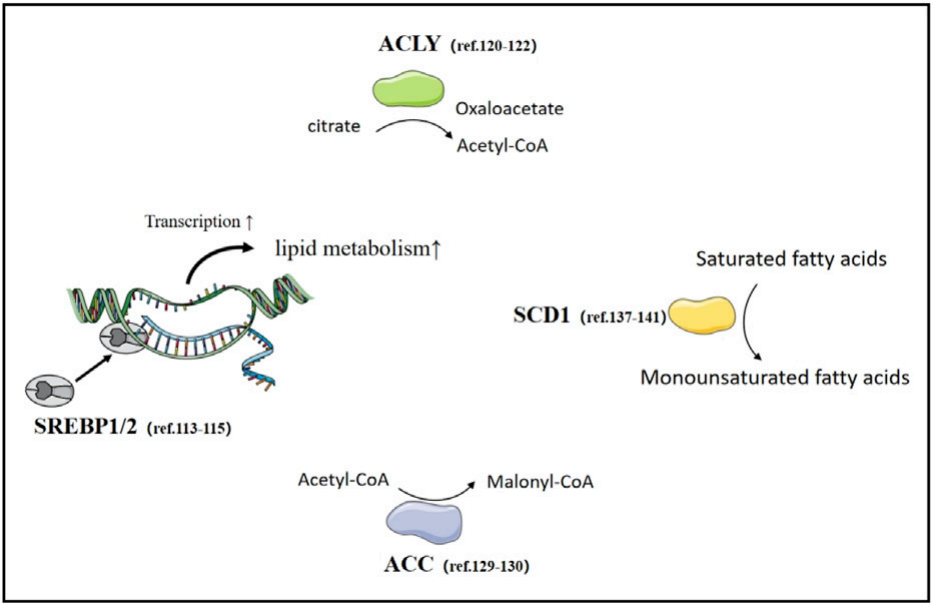
Fatty acid synthesis in lung Cancer. The important transcription factor SREBPs and rate-limiting enzymes in fatty acid synthesis, as well as relevant literature in lung cancer research.

Fatty acid synthesis (FAS) is an important metabolic process within cells, involving the conversion of acetyl-CoA and malonyl-CoA into long-chain fatty acids. This process occurs in the cytoplasm of cells, primarily taking place in the cytoplasm of eukaryotic and prokaryotic organisms. Fatty acid synthesis is crucial for physiological functions such as the construction of cell membranes, and signal transduction (Wedan et al., 2024; Bogie et al., 2020). The role of fatty acid synthesis in cancer cells is extremely important as it provides the necessary energy and biosynthetic precursors for the rapid proliferation of cancer cells. Compared to normal cells, cancer cells proliferate faster, require more energy, and their energy production is closely related to the metabolism of materials. Cancer cells tend to convert glucose into lactate through glycolysis. Nevertheless, fatty acid metabolism is closely related to tumor development, and the energy provided by fatty acids in cancer cells may promote the occurrence, development, and metastasis of tumors (La and Sr, 2021; Röhrig and Schulze, 2016; Currie et al., 2013; Ko et al., 2020) (Figure 4).

Sterol Regulatory Element-Binding Proteins (SREBPs) are a group of important transcription factors that regulate the endogenous synthesis of cholesterol, fatty acids, triglycerides, and phospholipids to maintain intracellular lipid balance. SREBP1 plays a particularly crucial role in this process, primarily responsible for regulating the synthesis of fatty acids (Shimano and Sato, 2017). Recent research indicates that the role of SREBPs in tumor is becoming increasingly significant (Cheng et al., 2022; Su and Koeberle, 2024; Cheng et al., 2015). Inhibiting SREBPs can affect the peroxidation of polyunsaturated fatty acids (Kawamura et al., 2022) which helps to overcome tumor resistance to therapy. The activity of SREBPs is regulated by various signaling pathways in tumor, including p53, Akt, ROS, microenvironmental pH, and so on (Kawamura et al., 2022; Rong et al., 2024; Liu et al., 2015; Yi et al., 2020). SREBP1 has been confirmed by numerous studies to be associated with the proliferation and invasion of lung cancer cells, among other biological behaviors (Jin et al., 2024; Liu et al., 2021). KRAS gene mutations are one of the main causes of lung adenocarcinoma, and the activation of KRAS in lung cancer can enhance the activity of SREBP through the ERK-mTORC1 pathway, thereby stimulating the synthesis of fatty acids (Gouw et al., 2017). Therefore, targeting SREBP-regulated fatty acid synthesis is a unique therapeutic target for KRAS-mutated lung cancer.

ATP citrate lyase (ACLY) is a key enzyme in the glycolipid metabolic pathway, catalyzing the breakdown of citrate into acetyl-CoA and oxaloacetate, thereby providing the necessary acetyl-CoA for the synthesis of fatty acids and cholesterol. Moreover, ACLY generates acetyl-CoA within the cell nucleus, a process essential for the acetylation of histones and the regulation of gene expression. By converting citrate into acetyl-CoA, ACLY supplies the necessary metabolic substrates for the proliferation and survival of cancer cells, thereby facilitating fatty acid synthesis and cholesterol biosynthesis. In cancer cells, the expression of ACLY is often upregulated, and it plays a significant role in the occurrence, development, and metastasis of tumors (Wen et al., 2019; Chen Y. et al., 2024; Granchi, 2018; Xiang et al., 2023). ARHGEF3, a member of the Rho GEFs family, is highly expressed in non-small cell lung cancer, enhancing the protein stability of ACLY, thereby promoting the proliferation of NSCLC cells both *in vitro* and *in vivo* (Zhou et al., 2022). In contrast, in the A549 lung cancer cell line, the inhibition of ACLY leads to growth arrest both *in vitro* and *in vivo* (Migita et al., 2008). This suggests that ACLY is a promising target for the treatment of non-small cell lung cancer (Dong et al., 2023).

Acetyl CoA carboxylase (ACC) is a biotin-dependent enzyme that plays a crucial regulatory role in fatty acid synthesis. ACC controls the rate of long-chain fatty acid entry into the mitochondria by inhibiting carnitine palmitoyltransferase 1 (CPT 1) through its production of malonyl CoA, which is the rate-limiting step for β-oxidation (Wang Y. et al., 2022). In cancer cells, the activity of ACC increases, promoting fatty acid synthesis, which is vital for the growth and survival of tumor cells (Chen G. et al., 2023; Svensson et al., 2016; Rios Garcia et al., 2017; Bacci et al., 2024). In the tumor microenvironment, ACC activity and expression may impact CD8^+^ T cell function. Inhibition of ACC in tumor microenvironment can enhance mitochondrial use of free fatty acids and bioenergetics, improving CD8^+^ T cell survival and antitumor effects in tumor microenvironment (Hunt et al., 2024). Due to its key role in tumor metabolism, ACC has become a potential target for cancer treatment. Targeting the metabolic pathways of ACC, such as by inhibiting its activity, may be effective for cancer treatment (Jsv et al., 2019). Studies have reported a novel lncRNA, CTD-2245E15.3, which promotes the occurrence of lung cancer by regulating the anabolic enzyme ACC1[(Wang C. et al., 2021)]. Non-small cell lung cancer cells rely on *de novo* fatty acid synthesis to promote growth, a process that requires ACC enzyme. Inhibiting ACC can restrict the growth of non-small cell lung cancer, suggesting that ACC inhibitors may become a new means of tumor treatment (Svensson et al., 2016). In addition, ACC is also associated with lung metastatic cancer, and relevant research data reveal a mechanism by which downregulation of ACC directs the formation of an immunosuppressive pre-metastatic niche (PMN) in the lungs for breast cancer (Huang et al., 2023). Therefore, ACC plays a significant role in the development of lung cancer and can become a very promising therapeutic target for lung cancer treatment.

Stearoyl-CoA desaturase-1 (SCD1), as a key enzyme, is responsible for converting saturated fatty acids into monounsaturated fatty acids, playing an essential role in maintaining the body’s metabolic and tissue homeostasis (Mauvoisin and Mounier, 2011; Sun et al., 2024). In cancer cells, the high activity of SCD1 is a crucial determinant of fatty acid composition, driving cancer cell metabolism towards more active lipogenesis and less lipid oxidation (Xuan et al., 2022). Furthermore, the activity of SCD1 also promotes cancer cell proliferation, survival, and invasiveness, partly by altering the lipid domains of the plasma membrane, which favors the activation of tyrosine kinase receptor signaling platforms (Sen et al., 2023; F et al., 2021; D et al., 2022). In lung cancer cell lines, upregulation of SCD1 expression is observed. Specifically, cells resistant to gefitinib have higher lipid droplet content and SCD1 expression levels than gefitinib-sensitive cells in non-small cell lung cancer cell lines and patient tissues (Q et al., 2019). A549 and H1573 cells with overexpressed SCD1 exhibit higher IC50 values for gefitinib, which is associated with the activation of the EGFR/PI3K/AKT signaling pathway and promotes the progression of lung tumors (She et al., 2019). Studies have confirmed that the expression of SCD1, enhanced by SNORD88C, promotes the proliferation and metastasis of non-small cell lung cancer both *in vitro* and *in vivo* (K et al., 2023). Additionally, a research team has demonstrated the involvement of SCD1 in the regulation of the Hippo pathway in lung cancer and pointed out that fatty acid metabolism is a key regulatory factor for lung cancer stem cells (Noto et al., 2017). Using CRISPR screening technology, scholars have confirmed that SCD1 is particularly important in STK11/KEAP1 co-mutated lung adenocarcinoma, representing a selective vulnerability. Genetic and pharmacological inhibition of SCD1 enhances the ferroptosis induced by erastin and RSL3 [(Wohlhieter et al., 2020)], further highlighting the role of SCD1 in the development of lung cancer and its status as a key regulatory factor in lung cancer stem cells.

### 4.3 Fatty acid oxidation

Fatty Acid Oxidation (FAO) is an important process in cellular metabolism, involving the breakdown of fatty acids into energy-rich molecules such as Acetyl-CoA, which can further enter the citric acid cycle to produce a large amount of ATP, providing energy for the cell (Grabner et al., 2021; Lopaschuk et al., 2010). The rate-limiting enzyme for fatty acid oxidation is Carnitine Palmitoyltransferase 1 (CPT1). CPT1 is a key enzyme in the process of fatty acid oxidation, catalyzing the activation of long-chain fatty acids and transporting them into the mitochondria for beta-oxidation. Fatty acid oxidation plays a significant role in the metabolic reprogramming of cancer cells, providing not only an energy source for cancer cells but also participating in the occurrence, development, metastasis, immune evasion, and response to treatment of tumors (Carracedo et al., 2013; Y et al., 2018; Li Y-J. et al., 2022).

Carnitine Palmitoyltransferase 1 (CPT1) is a key rate-limiting enzyme in the process of fatty acid oxidation, playing an essential role in carnitine-dependent transport across the mitochondrial inner membrane. A deficiency in CPT1 leads to a decrease in the rate of fatty acid β-oxidation. Inhibition of CPT1 has been shown to suppress tumor growth, and CPT1 is overexpressed in various types of cancer, where it regulates gene expression and apoptosis in tumor cells. In the tumor microenvironment, CPT1 also plays a significant role in tumor neovascularization (Wang et al., 2018; Qu et al., 2016; Yang H. et al., 2021; Schlaepfer et al., 2014). A study shows that CPT1A significantly affects ferroptosis resistance in lung cancer stem cells and drives complex metabolic reprogramming. Additionally, a new feedback regulatory mechanism between CPT1A and c-Myc in the ferroptosis process of lung cancer stem cells was discovered, revealing how metabolic rewiring supports tumor cell survival during immune clearance (Ma et al., 2024).

## 5 Amino acid metabolism in lung cancer

Amino acids are not only the basic building blocks of proteins, but also play a crucial role in numerous biological processes such as cell structure construction, enzyme catalysis, signal transduction, and immune responses. Disorders in their metabolism are closely related to the occurrence and development of various diseases (Paulusma et al., 2022; Cibrian et al., 2020). In oncology, the role of amino acid metabolism is particularly significant, as tumor cells alter the uptake and metabolism of amino acids to meet their proliferative needs (Butler et al., 2021). Amino acids not only provide energy and biosynthetic precursors for the rapid growth of tumor cells (Vettore et al., 2020; Qiu et al., 2023), but also the high expression of certain amino acid metabolic enzymes, such as asparaginase, which plays a role in the treatment of leukemia, and the enhancement of branched-chain amino acid (BCAA) metabolism, may promote the migration and invasion of tumor cells (Qian et al., 2023). Furthermore, amino acid metabolism shapes the tumor microenvironment and affects the function of immune cells, which may have an enhancing or inhibitory effect on immune surveillance (Yang et al., 2023). Therefore, amino acid metabolism has a profound impact not only on the biological behavior of tumor cells but also regulates immune responses within the tumor microenvironment.

### 5.1 Glutamine metabolism

Glutamine, a non-essential amino acid, is the most abundant free amino acid in the human body and plays a critical role in various metabolic processes. During periods of stress, illness, or injury, the body’s requirements for glutamine can exceed its production capacity, making it conditionally essential (Yoo et al., 2020; Wang et al., 2019; H and ussinger, 1990; Bodineau et al., 2022). Glutamine plays a central role in tumor cell metabolism; it is not only a key energy source for tumor growth and proliferation but also participates in maintaining the redox balance within tumor cells (Altman et al., 2016). In tumor cells, the expression levels of certain enzymes related to glutamine metabolism are significantly higher than in normal cells. For instance, high expression levels of glutamine synthetase and glutaminase are closely associated with the severity of the tumor and poor prognosis (Jin et al., 2023). Moreover, glutamine metabolism also affects the function of immune cells in the tumor microenvironment, which may enhance or suppress the action of immune surveillance (Edwards et al., 2021; Wang et al., 2024). In the tumor microenvironment, glutamine metabolism is involved in regulating immune responses, and its metabolic products can modulate the activity of immune cells, thereby affecting their ability to attack the tumor (Leone et al., 2019). Therefore, glutamine metabolism is not only crucial for the survival of tumor cells but also has a significant impact on immune responses within the tumor microenvironment. The growth of lung cancer cells largely depends on the metabolism of glutamine (Kim et al., 2022; T et al., 2022). In particular, cancer cells with mutations in Keap1 or Nrf2 require enhanced glutamine catabolism to maintain their growth (Romero et al., 2017). These cells alleviate oxidative stress by increasing the conversion of glutamine to glutamate and subsequently synthesizing glutathione, thereby supporting the proliferation and survival of lung cancer cells (Han et al., 2024). Moreover, blocking glutamine metabolism not only enhances the anti-cancer immune response, but glutamine is also essential for the development and activation of effector T cells in the tumor microenvironment that have anti-tumor functions (Huang et al., 2022). Inhibitors of glutaminase may suppress the CD8 T cells activated by anti-PD-1 immunotherapy, indicating that the impact of glutamine metabolism should be carefully considered when designing anti-cancer strategies (Best et al., 2022).

### 5.2 Argine metabolism

Arginine, owning a central role in the urea cycle by aiding the liver in converting toxic ammonia into urea, is an essential amino acid for human health, which is the primary route for mammals to eliminate nitrogenous waste (Mangoni et al., 2019). Moreover, arginine is also crucial for reproductive health, as its deficiency may lead to reduced sperm production and thus affect fertility (Azhar et al., 2023). Arginine plays a crucial role in tumor metabolism and is closely associated with the development of cancer. In liver cancer, despite the reduced expression of arginine synthesis genes, tumor cells accumulate higher levels of arginine by enhancing its uptake and decreasing its conversion to polyamines. This accumulation of arginine further promotes tumor formation and triggers a series of metabolic reprogramming, including changes in glucose, amino acid, nucleotide, and fatty acid metabolism (Mossmann et al., 2023). Moreover, arginine metabolism, particularly the metabolism of branched-chain amino acids, may play a role in tumor progression, with increased metabolic activity potentially promoting the migration and invasion of tumor cells (Sciacovelli et al., 2022). Additionally, the inhibition of glutaminase has a negative impact on CD8 T cells activated by anti-PD-1 immunotherapy, indicating that the blockade of arginine metabolism might affect the efficacy of immunotherapy (Canale et al., 2021; Tharp et al., 2024). Arginine plays a crucial role in enhancing the proliferation, invasion, and metastatic capabilities of non-small cell lung cancer (Chen D. et al., 2023). In MYC-driven small cell lung cancer, tumors rely heavily on the arginine synthesis pathway, and depleting arginine using pegylated arginine deiminase can significantly inhibit tumor growth and improve survival rates in mouse tumor models (Chalishazar et al., 2019). In non-small cell lung cancer with KRAS mutations, the expression of arginine succinate synthetase is silenced, leading to a dependence on the arginine transporter SLC7A1 for arginine uptake from the extracellular environment. Thus, SLC7A1-mediated arginine uptake becomes a potential therapeutic vulnerability for the treatment of KRAS-mutant non-small cell lung cancer (Gai et al., 2024).

### 5.3 Serine metabolism

Serine is a non-essential amino acid that participates in a variety of biosynthetic processes, including the synthesis of purines, pyrimidines, and phospholipids, which are crucial for the construction and function of cell membranes. Restricting the intake of serine can suppress the growth of cancer cells by promoting the synthesis of toxic sphingolipids (Muthusamy et al., 2020). Relevant studies have shown that restricting serine and glycine in the diet significantly slows down the growth of intestinal cancer in mice. Colon cancer cells promote metastasis by enhancing the activity of key enzymes in serine synthesis, a process closely related to the generation of S-adenosylmethionine (Zhang Y. et al., 2021). Inhibiting serine metabolism in lung cancer cells leads to a decrease in cellular proliferation both *in vitro* and *in vivo* (He et al., 2022). In non-small cell lung cancer with wild-type IDH, increased serine synthesis disrupts the balance between glutathione and reactive oxygen species, supports pyrimidine biosynthesis, maintains tumor-initiating capacity, and enhances resistance to gemcitabine chemotherapy (Zhang et al., 2023). Furthermore, NRF2, a regulator of serine metabolism in non-small cell lung cancer, is associated with the invasiveness of lung cancer (Gm et al., 2015). Overall, the significance of serine in tumor metabolism offers new perspectives for cancer treatment.

### 5.4 Cystine metabolism

**FIGURE 5:**
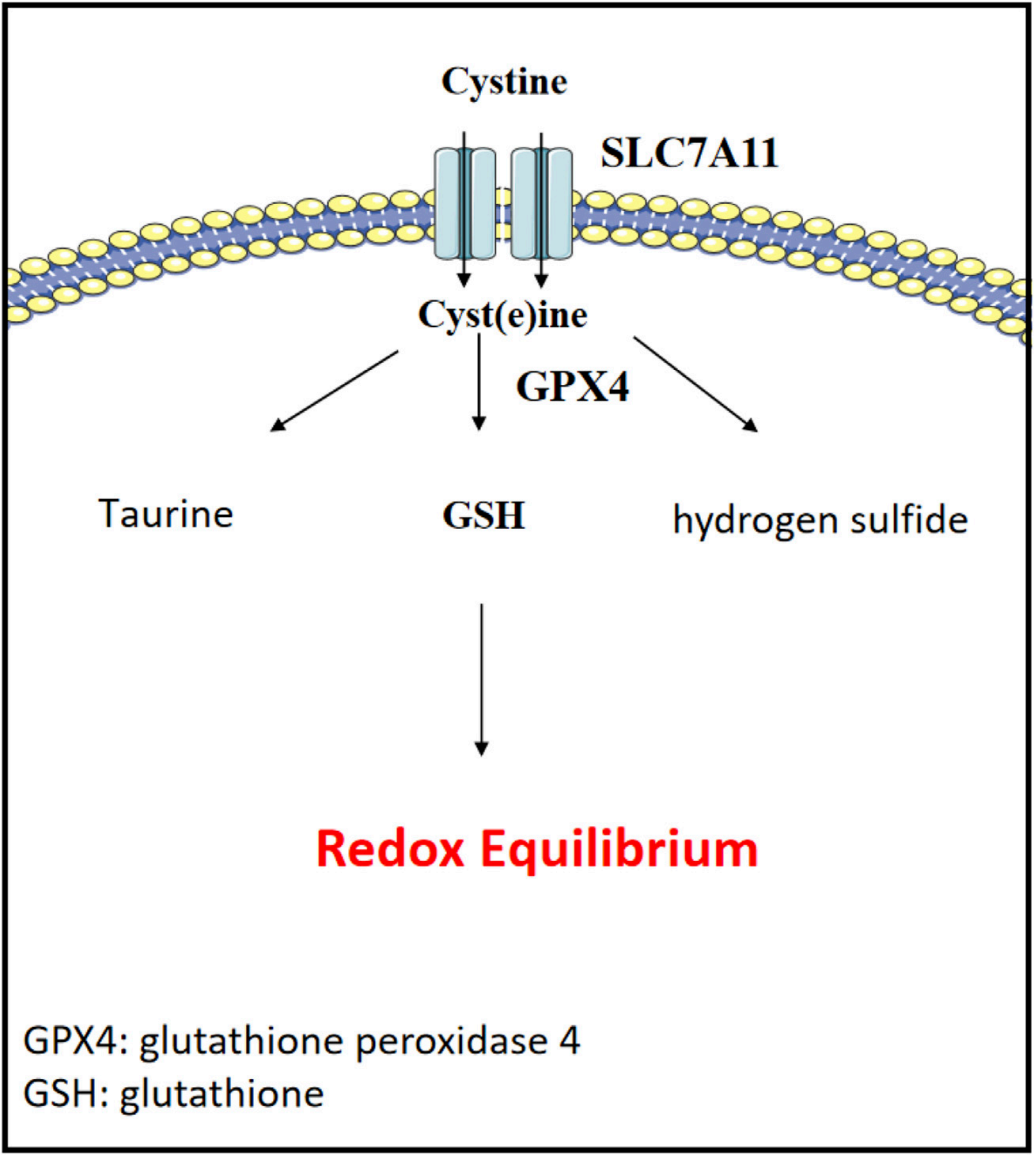
Cystine metabolism and redox equilibrium. Cystine is vital for redox regulation in cancer metabolism and cell survival.

Cystine is a sulfur-containing amino acid formed by the oxidation of two cysteine molecules linked together by a disulfide bond. It is one of the important amino acids in the human body, known for its roles in enhancing immune function, promoting growth and development, improving liver function, and providing antioxidant effects (Figure 5). In cancer metabolism and cancer cell survival, cystine plays a crucial role in the redox regulation of cellular status and protein function. The cystine/cysteine redox cycle effectively protects cells from oxidative stress-induced cell death or ferroptosis by increasing intracellular cysteine levels and maintaining very high extracellular cysteine concentrations (Badgley et al., 2020; Liu et al., 2020; Koppula et al., 2021b). Through metabolomic approaches, related studies have found that mutated and activated KRAS significantly increases intracellular cysteine levels and glutathione biosynthesis (Hu et al., 2020). The ablation of RBMS1 inhibits the translation of SLC7A11, reduces SLC7A11-mediated cysteine uptake, and promotes ferroptosis in lung cancer cells (Zhang W. et al., 2021). SOX2 also activates SLC7A11 in lung cancer cells, enhancing their cysteine uptake (Wang X. et al., 2021).

## 6 Application of targeting metabolism in lung cancer

### 6.1 Targeting glucose metabolism

2-Deoxyglucose (2-DG) is converted into 2-deoxyglucose-6-phosphate by the catalysis of hexokinase, a product that cannot be further metabolized by cells. As it cannot be broken down further, it accumulates within the cells and competitively binds to hexokinase, thereby inhibiting its activity and slowing down the glucose uptake process. Additionally, 2-DG increases oxidative stress, inhibits N-glycosylation, and induces autophagy. It can be combined with other therapeutic agents or radiotherapy to exhibit synergistic anticancer effects (Zhang et al., 2014). Lung cancer cells treated with 2-deoxyglucose show a significant decrease in cell viability, and this cytotoxicity may be closely related to the lung cancer genes LKB1 and IGF1R (Inge et al., 2009; Liu et al., 2018). The combination of 2-DG with other effective drugs for the treatment of lung cancer exhibits better toxicity than the use of compounds alone (Hou et al., 2016; Guo et al., 2024).

3-bromopyruvate is a small and highly reactive molecule that targets the overexpressed monocarboxylate transporters in cancer cells, leading to tumor cell death by depleting ATP through the inactivation of glycolysis and mitochondrial energy production pathways. 3-bromopyruvate can significantly inhibit the proliferation of A549 cells and induce apoptosis (Cunha et al., 2022; Dai et al., 2019).

### 6.2 Targeting lipid metabolism

The fatty acid synthase inhibitor TVB-2640 has shown strong anti-tumor effects and has demonstrated good therapeutic efficacy and manageable safety in clinical trials (Falchook et al., 2021). TVB-2640 significantly inhibited the lipogenic phenotype in lung cancer cells, and weakened the capabilities of self-renewal, chemoresistance, and tumorigenesis mediated by USP13 in small cell lung cancer (Wang J. et al., 2022).

Etomoxir is an irreversible inhibitor of carnitine palmitoyltransferase 1a (CPT-1a), which inhibits fatty acid oxidation by suppressing CPT-1a. By inhibiting lipolysis with Etomoxir, it can synergistically suppress the proliferation of drug-resistant lung adenocarcinoma cells (Li et al., 2013).

ND-646 is an inhibitor of acetyl-CoA carboxylase (ACC), which reduces fatty acid synthesis by inhibiting ACC1 and ACC2. In the non-small cell lung cancer cell line A549, ND-646 can decrease the production of palmitic acid and total fatty acid content within the cells, induce apoptosis, and trigger endoplasmic reticulum stress (Li et al., 2019). In a mouse xenograft model of A549 lung cancer, when administered at a dose of 25 mg/kg twice daily, ND-646 was able to inhibit tumor growth (Jsv et al., 2019).

### 6.3 Targeting acid amino metabolism

CB-839, also known as Telaglenastat, is a first-in-class, selective, reversible, and orally active glutaminase 1 inhibitor. In lung cancer cells, CB-839 can leads to increased levels of intracellular reactive oxygen species (ROS) and decreased levels of ATP, NADPH/NADP + ratio, and glutathione, thereby increasing energy and redox stress (Galan-Cobo et al., 2019).

NCT-503 is a phosphoglycerate dehydrogenase (PHGDH) inhibitor. PHGDH catalyzes the first step in the *de novo* synthesis of L-serine from 3-phosphoglycerate. It has been identified as an inhibitor of PHGDH and was found to be inactive against a panel of other dehydrogenases and showed minimal cross-reactivity in a panel of G-protein-coupled receptors. In lung cancer, the combination of PKM2-IN-1 and NCT-503 has shown significant anti-cancer effects by inducing G2/M cell cycle arrest and apoptosis (Wang K. et al., 2023).

## 7 Conclusion

Lung cancer is a globally common malignant tumor, with both incidence and mortality rates remaining high. The treatment of lung cancer requires the joint efforts of a multidisciplinary team, including surgery, radiotherapy, chemotherapy, targeted therapy, and immunotherapy. Despite the development of targeted and immunotherapies offering new hope for the treatment of lung cancer, it remains a clinical challenge that is difficult to completely cure. Therefore, the development of new treatment strategies is one of the key focuses of current lung cancer research.

The metabolic reprogramming of lung cancer cells is a key driver of tumor development, involving significant changes in energy metabolism and biosynthetic pathways. These changes include glycolysis, amino acid metabolism, fatty acid metabolism, and more. These metabolic alterations not only support the rapid proliferation of tumor cells but also affect the tumor microenvironment and immune response. For example, lung cancer cells may meet energy demands by enhancing the glycolytic process, even under conditions of sufficient oxygen, a phenomenon known as the “Warburg effect.” Additionally, lung cancer cells may alter fatty acid synthesis and oxidation to meet their needs for energy and biosynthetic precursors.

The document also discusses the role of glucose transport proteins (such as GLUTs), glycolysis-related enzymes (such as hexokinase 2 and lactate dehydrogenase A), fatty acid metabolism-related proteins (such as CD36 and fatty acid-binding proteins), and some amino acids and their metabolic pathways in lung cancer. The expression levels of these proteins are often upregulated in lung cancer and are closely related to tumor proliferation, invasion, and prognosis. Targeted therapies against these metabolic pathways, such as inhibiting specific metabolic enzymes, may provide new opportunities for lung cancer treatment.

The manuscript in question primarily focuses on the three major metabolic pathways—glucose, lipid, and amino acid metabolism—which are indeed the most extensively studied and form the cornerstone of current research in the field. However, we acknowledge that this concentration inherently limits the scope of our review, as it does not encompass other significant metabolic pathways that may also play crucial roles in the development and progression of lung cancer. This omission is a recognized limitation of our study, and we hope that future research will address these gaps by delving into the less-explored metabolic avenues. We anticipate that with more articles dedicated to these areas, a more comprehensive understanding of the metabolic landscape in lung cancer can be achieved, ultimately contributing to the advancement of therapeutic strategies and patient outcomes.

Overall, this document provides a comprehensive perspective on how lung cancer cells adapt to their growth and survival needs through metabolic reprogramming and explores how these metabolic changes can serve as potential therapeutic targets. By gaining a deeper understanding of the metabolic characteristics of lung cancer, researchers can develop more effective treatment strategies to improve the prognosis of lung cancer patients.
